# Characterization of Labeled Gold Nanoparticles for Surface-Enhanced Raman Scattering

**DOI:** 10.3390/molecules27030892

**Published:** 2022-01-28

**Authors:** Fahad M. M. Aldosari

**Affiliations:** School of Physics and Astronomy, University of Exeter, Exeter EX4 4QL, UK; Fa301@exeter.ac.uk

**Keywords:** SERS, gold nanoparticles, Raman, absorption, surface plasmons, reporter, vibrational spectroscopy, imaging

## Abstract

Noble metal nanoparticles (NP) such as gold (AuNPs) and silver nanoparticles (AgNPs) can produce ultrasensitive surface-enhanced Raman scattering (SERS) signals owing to their plasmonic properties. AuNPs have been widely investigated for their biocompatibility and potential to be used in clinical diagnostics and therapeutics or combined for theranostics. In this work, labeled AuNPs in suspension were characterized in terms of size dependency of their localized surface plasmon resonance (LSPR), dynamic light scattering (DLS), and SERS activity. The study was conducted using a set of four Raman labels or reporters, i.e., small molecules with large scattering cross-section and a thiol moiety for chemisorption on the AuNP, namely 4-mercaptobenzoic acid (4-MBA), 2-naphthalenethiol (2-NT), 4-acetamidothiophenol (4-AATP), and biphenyl-4-thiol (BPT), to investigate their viability for SERS tagging of spherical AuNPs of different size in the range 5 nm to 100 nm. The results showed that, when using 785 nm laser excitation, the SERS signal increases with the increasing size of AuNP up to 60 or 80 nm. The signal is highest for BPT labelled 80 nm AuNPs followed by 4-AATP labeled 60 nm AuNPs, making BPT and 4-AATP the preferred candidates for Raman labelling of spherical gold within the range of 5 nm to 100 nm in diameter.

## 1. Introduction

Gold nanoparticles (AuNPs) are noble metal particles with variable optical characteristics, making them unique nanostructures for sensing, imaging, and medication targeting. Gold is one of the few noble metals with an optical property due to its high interaction with electromagnetic radiation in the visible part of the spectrum. It absorbs and scatters light simultaneously as it interacts with it. The amplified oscillation of the metal’s electron system occurs because the frequency of the absorbed light overlaps with the oscillation frequency of the electrons [1]. As a result, surface plasmons produce an electromagnetic field on the nanostructured metal surface. The dispersed light may be captured for imaging purposes while the absorbed light is converted to heat by surface plasmons. Surface plasmon generation and the quantity of light scattered are greatly influenced by particle size, shape, aggregation state, composition, and the dielectric constant of the surrounding medium. Carbon materials play an important role in surrounding the magnetic nanoparticles due to their electric properties, chemical/thermal stability, abundant resources, and facile manipulation [2]. UV/visible spectroscopy can readily monitor the surface plasmon resonance (SPR) wavelength.

The exotic optical properties and intriguing morphologies of organic nanoparticle clusters have attracted wide attention [3]. Raman spectroscopy provides label-free chemically specific information about biological samples under characteristic vibrational modes in molecules for a wide range of biomedical research applications [4]. However, the Raman scattering effect is fragile, and this accounts for low-intensity Raman signals in most cases. Surface-enhanced Raman scattering (SERS) can overcome the drawback of reduced sensitivity by exploiting the plasmon resonance at noble metal surfaces [5]. SERS spectroscopy relies on enhancing Raman scattering signals from molecules near active nanostructures. The mechanism relevant to SERS is the surface enhancement of the electromagnetic field at the interface between the SERS active substrate (e.g., a noble metal such as gold or silver) and a molecule of interest through resonant excitation of surface plasmons of the metal.

When combined with a given nanomaterial, Raman spectroscopy is more effective in detecting and mapping cancer cell models [6]. For many years, scientists in multiple research areas of physics, chemistry, material science, and life sciences have applied the surface-enhanced Raman scattering (SERS) technique to detect specific molecules present in small concentrations in biological media. SERS has several advantages over traditional vibrational spectroscopy techniques as it has enhanced molecular sensitivity, selectivity, and accuracy. Sensitivity in SERS spectroscopy is improved by amplifying Raman signals of biomolecules in the vicinity of active surfaces such as silver, gold, or nanostructures (nanofibres, nanowires, nanowhiskers, nanorods, and nanostars). The marked signal enhancement of the order of 10^4^–10^10^ that originates from the localized surface plasmon resonance (LSPR) can allow single-molecule detection of species near the nanomaterial’s active surface. Therefore, specific Raman signatures with strong sensitivity can effectively be disentangled from the much weaker spectrum of other non-proximal molecules. SERS-based techniques have been used in affordable, field-transferable, qualitative, and quantitative detection of biomarkers [7,8]. In prostate tumor cells, nanoparticles have been deployed to enable a viable SERS-based method for differentiating tumor and regular cell lines [9].

According to Szekeres and Kneipp, [10] the formation of nanoaggregates which can significantly augment the SERS detection, can be impaired due to multilayer protein adsorption (protein “corona”) and high viscosity on the surface of the nanoparticles for providing high SERS response when estimating cellular protein concentrations. In this regard, further positioning and development of intracellular aggregates can produce high SERS signals in live cells. Gold nanoparticles (AuNPs) become optimal when their size is around 50 nm in diameter due to their critical surface concentration or area. Furthermore, minimum toxicity has been shown for AuNPs of 50 nm size with biomolecules at biological concentrations [11]. Therefore, introducing a minimum amount of gold can be suitable for detecting essential biomolecules at biological concentrations when using SERS with the lowest possible toxicity [12]. Precisely, the advantages of SERS-active nanoparticles include providing higher sensitivity, unparalleled multiplexing abilities, and accurate signal specificity compared to conventional imaging models [13].

AuNPs employed in in-vitro cell investigations are typically synthesized using wet-synthesis techniques in the 2–100 nm size range [14,15]. Typically, a reducing agent such as trisodium citrate or sodium borohydride is used. It is critical to utilize a nontoxic reducing agent because the objective is to use them in living cell research. To generate rod-shaped AuNPs, cetyltrimethylammonium bromide (CTAB) is utilized, although it is hazardous to the living cell [16]. AuNPs are employed in various ways, including as-synthesized and following surface modification. Surface modification aims to lower toxicity, attach functional groups or coatings for targeting or distribution, or both [17,18]. Since AuNPs are permitted to interact with living cells by being added to cell culture, the surface chemistry, size, and shape of the AuNPs and their absorption mechanism should all be carefully studied for a minimal harmful impact on cells.

Previous works have investigated the LSPR and SERS activity of AuNPs in a similar range of sizes as studied here, using rhodamine 6G [19], malachite green isothiocyanate [20], or oxalate salt [21], but crucially not with sets of different Raman labels to single out optimal candidates (s) for SERS tagging. The present study is aimed to characterize colloidal AuNPs of different sizes and with various Raman labels or reporters using transmission electron microscopy (TEM), UV-visible absorbance, dynamic light scattering (DLS), and Raman micro-spectroscopy. Results are discussed, and a selection is dedicated to the AuNP candidates to be used in further SERS applications regarding nanotheranostics.

## 2. Materials and Methods

### 2.1. Labeled Gold Nanoparticles

All compounds, additives, and solvents which include 5, 10, 15, 40, 60, 80, and 100 mm diameter bare spherical gold nanoparticles (NanoXact, nanoComposix, 0.05 mg/mL in aqueous 2 mM sodium citrate), 4-mercaptobenzoic acid (4-MBA) (Sigma-Aldrich, 99% pure) (St. Louis, MO, USA), 2-naphthalenethiol (2-NT) (Sigma-Aldrich, 99% pure), 4-acetamidothiophenol (4-AATP) (Sigma-Aldrich, 95% pure), biphenyl-4-thiol (BPT) (Sigma-Aldrich, 97% pure), and ethanol (Sigma-Aldrich, ≥99.5% pure) were used as received. Distilled water was obtained in our laboratory (ELGA Vision 250).

In this study, each Raman label’s 100 mM stock solution was prepared by adding 15.4 mg, 16 mg, 16.7 mg, or 18.6 mg of 4-MBA, 2-NT, 4-AATP, or BPT, respectively, to 1 mL ethanol. Each solution was mixed in a Vortex Varimix shaker (SciQuip) and diluted 1:99 in ethanol to make up a 1 mM label solution. Then 100 µL of label solution was added to 1 mL AuNPs to a final label concentration of 9.1 × 10^−5^ M. The labeled AuNP solution was then centrifuged using a Hettich Mikro 22 centrifuge for 10 to 30 min, after shaking by hand for 5 min and vortexing for 1 min. The supernatant was then carefully removed, and the pellet was re-suspended in distilled water. Table 1 lists the centrifugation speeds used with all NP solutions; higher speeds were required to mix smaller AuNP solutions to obtain a pellet effectively.

**Figure 1:**
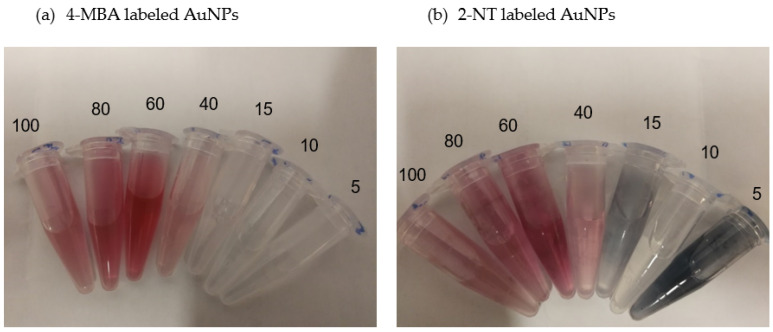

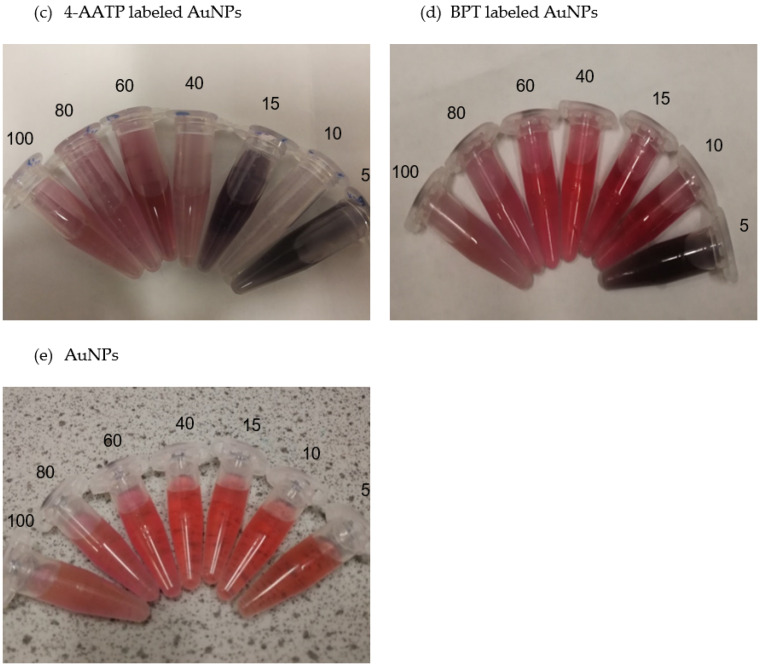
Solutions of AuNPs labeled with (**a**) 4-MBA, (**b**) 2-NT, (**c**) 4-AATP, (**d**) BPT, and (**e**) unlabeled AuNPs. Numbers indicate the particle size. Dark solutions in (**b**–**d**) denote aggregation for small-sized NPs, 15 nm and 5 nm.

Each solution was then transferred into a 2 mL Eppendorf tube (Figure 1) and stored for approximately 12 h at 4 °C in darkness before characterization.

### 2.2. TEM, UV-Vis, and DLS Analysis

The size and morphology of labeled AuNP samples were investigated using transmission electron microscopy (TEM) with a TEM-JEOL 2100 instrument at an operating voltage of 200 kV. Before the measurements, 2 drops of each solution were deposited onto a 200 mesh holey C-coated copper grid and left to dry in an oven at 80 °C for 4 h.

A volume of 1 mL AuNP solution was transferred into a quartz cuvette with a 1 cm path length. A UV-visible absorbance spectrum was acquired using a Thermo Scientific Evolution Array UV-visible spectrophotometer (Thermo Fisher Scientific) (Waltham, MA, USA). Spectra were obtained in the range 185–1100 nm, with 30 scans and 1000 ms integration time. The wavelength of maximum absorbance and shape of the localized surface plasmon resonance (LPSR) were analyzed using OriginPro software.

A Malvern Zetasizer Ultra running DTS software and 4 mW He−Ne fv at 633 nm was used for performing DLS measurements. A constant temperature of 25 °C was adjusted for the analysis at 173° and 90° scattering angles. A zeta potential cell was used for measuring zeta potential, whereas size was measured through disposable cuvettes of 1 cm path length. Data were collected in three phases and presented in the form of median and average values.

### 2.3. Raman Micro-Spectroscopy

The AuNP solutions were transferred onto a quartz Hellma 96-well microplate (volume of each well is 300 µL), and measurements were conducted using a Renishaw inVia Raman microscope. The system comprises two NIR diode lasers (785 nm and 830 nm), various objectives (5×, 10×, 20×, and 50×), and three diffraction gratings (300, 600, and 1200 L/mm). A motorized xyz stage was used to control and change the sample position. Spectra were acquired at room temperature (21 ± 2 °C) using both 785 nm and 830 nm excitations, the 600 L/mm grating and 50× objective (NA 0.75) in the range 283–2493 cm^−1^ at full laser power (20–30 mW at the sample), with 10 s acquisition time and 16 accumulations per spectrum. As a concentration gradient is expected for the solution in each well, depth-resolved Raman measurements were conducted (along the *z*-axis) starting from the surface and then down 100, 200, 300, 400, 500, and 600 µm into the solution. The most significant signals were observed for 600 μm depth, and hence these spectra were retained for analysis. WiRE 4.1 software was used for data acquisition and handling.

## 3. Results

**Figure 2:**
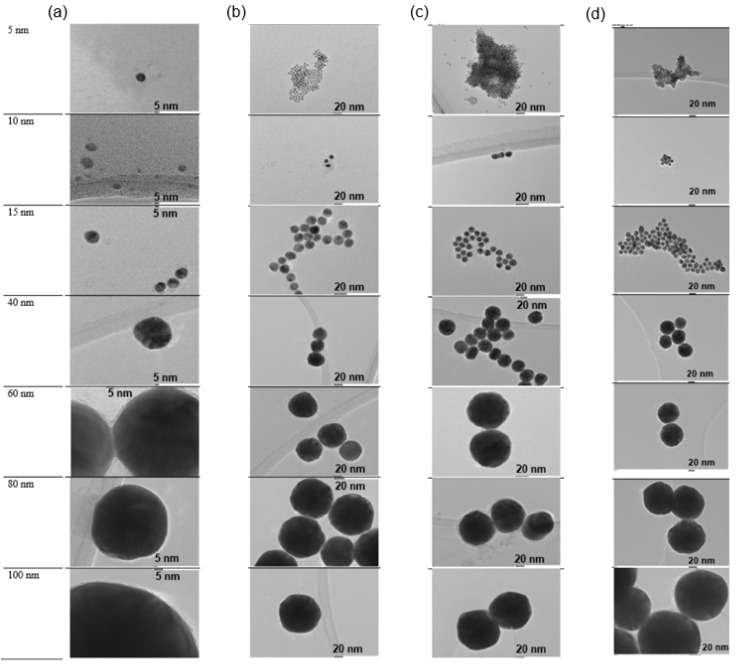
TEM images of (**a**) 4-MBA, (**b**) 2-NT, (**c**) 4-AATP, and (**d**) BPT labeled AuNPs of different sizes ranging between 5 nm and 100 nm. Scale bar: 5 nm in (**a**); 20 nm in (**b**–**d**).

It can be seen that the AuNPs are spherical, and the label produces some gray shadowing on the nanoparticle surface (Figure 2).

**Figure 3:**
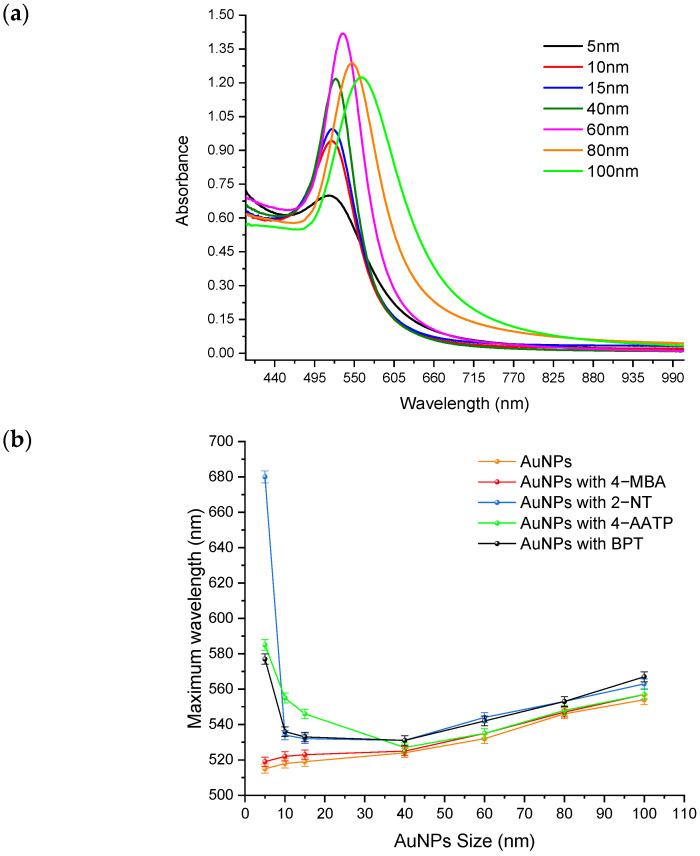
(**a**) UV-visible absorbance spectra of colloidal AuNPs of different sizes ranging between 5 nm and 100 nm. (**b**) The localized surface plasmon resonance maximum wavelength vs. AuNP size plot for all the solutions.

Figure 3a illustrate the UV-visible absorbance spectra of the bare AuNP solutions showing the LSPR shifts with increasing NP size, as expected for these solutions. Figure 3b is a plot of the LSPR maximum wavelength versus AuNP size for all the answers in this study, which shows that Raman intensity increase with the increase in the size of AuNPs. Compared to 4-MBA and 4-AATP, 2-NT and BPT labeling generate the highest redshift (14 nm) in LSPR. More significant shifts were seen between 5–15 nm due to aggregation of 2-NT, 4-AATP, and BPT tagged AuNPs.

For mono-disperse colloids, UV-Vis spectroscopy measurements were also carried out. FV The geometric size of the NPs was determined using AATP and BPT deposited on the surface, ensuring that the findings from these two approaches are comparable. In the instance of the DLS approach, the hydrodynamic size was measured. The ball model, which has the same diffusion coefficient as a measured NP, was used to classify this size. The size of the measured NPs can differ from that of the AATP or BPT procedures. In general, each offered measuring method demonstrates that the studied colloids only include monodisperse particles.

**Figure 4:**
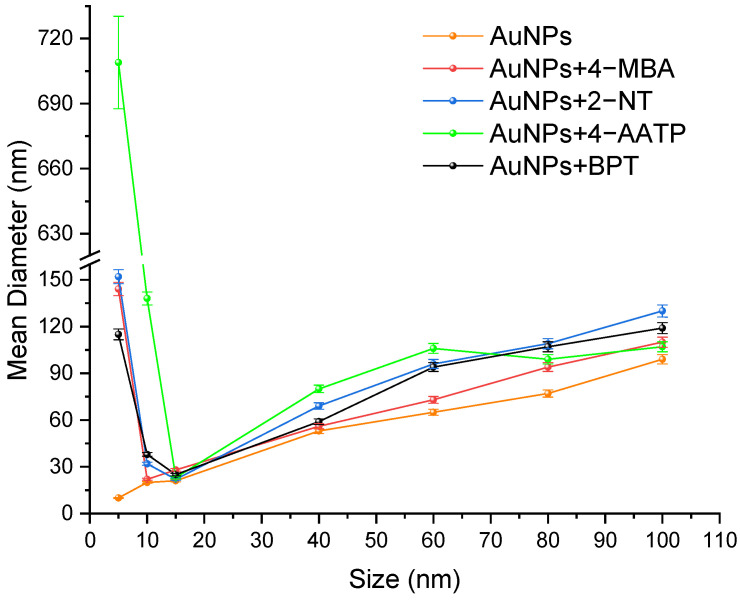
The DLS-derived AuNP size vs. nominal size plot for all the solutions. Error bars: standard deviation.

Figure 4 illustrate the results of DLS measurements of all AuNP solutions. The measured particle sizes essentially reproduce the nominal sizes of AuNPs. The mean diameter of 5 nm AuNps+4-MBA was measured to be 150 nm. In contrast, the particle sizes are more significant in the presence of a label, especially with 2-NT and BPT labels that have two benzene rings. Considering the AuNPs+4-MBA, the sample is transparent at a size of 5 nm as observed through the naked eye. However, there is the chance of a decreased concentration of gold nanoparticles because of a certain degree of aggregation in some of the samples.

It has been observed that the signal from small particles decreases when the volume of more considerable particles increases. This is an outcome of the fact that the competence of a particle for scattering light is proportional to its diameter to the sixth power. The peak coming from 10 nm AuNPs disappears entirely in the colloid at 95% of the content of these particles. The intensity of light scattered by larger particles (40 nm or 100 nm AuNPs) covers the signal from smaller ones (5 nm AuNPs). In particular, such an outcome wrongly suggests that merely monodisperse particles 40 nm (or 100 nm) in size are present in the colloid. The nanoparticles possess unique chemical and physical properties based on their size and surface area. The size determines their optical properties that impart different colors based on the absorption taking place in visible light. This is the reason why DLS value increased and then decreased for AuNPs when the size increased from 5 nm to 15 nm.

**Figure 5:**
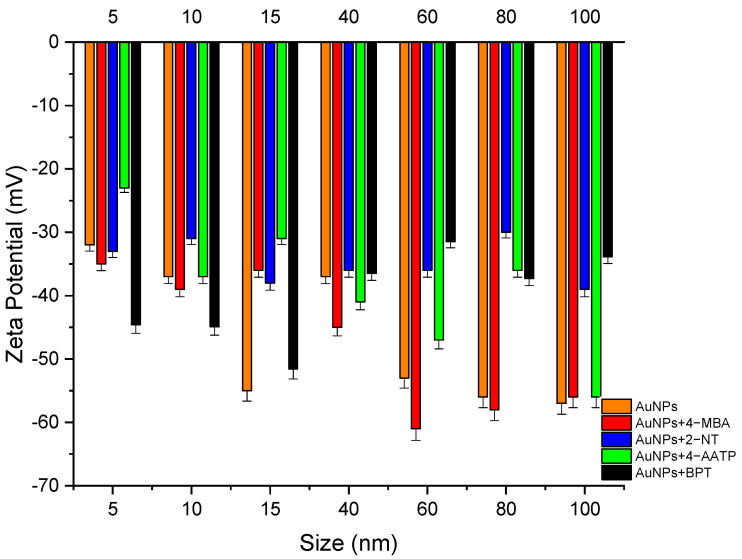
Zeta potential of (orange) bare AuNPs, and in the presence of a label: (red) 4-MBA, (blue) 2-NT, (green) 4-AATP, and (black) BPT for different NP sizes. Error bars: standard deviation.

The gold nanoparticles’ surface charge (zeta potential) before and after adding Raman reporters was negative, and the highest charge was observed for 60 nm AuNPs labeled with 4-MBA (−61 mV), as shown in Figure 5. It should be noted that the size distribution was closest to the size by the number of the nanoparticles. The Z average of the AuNPs was increased through surface charge, which indicates that DLS-derived AuNPs were successfully attached to the AuNP surface. Zeta potential measurement help to determine the stability of gold nanoparticles. It is demonstrated that the interaction of nanoparticles is affected by effective surface charge, along with the capillary wall and other nanoparticles. The mobility of nanoparticles is affected by these interactions.

**Figure 6:**
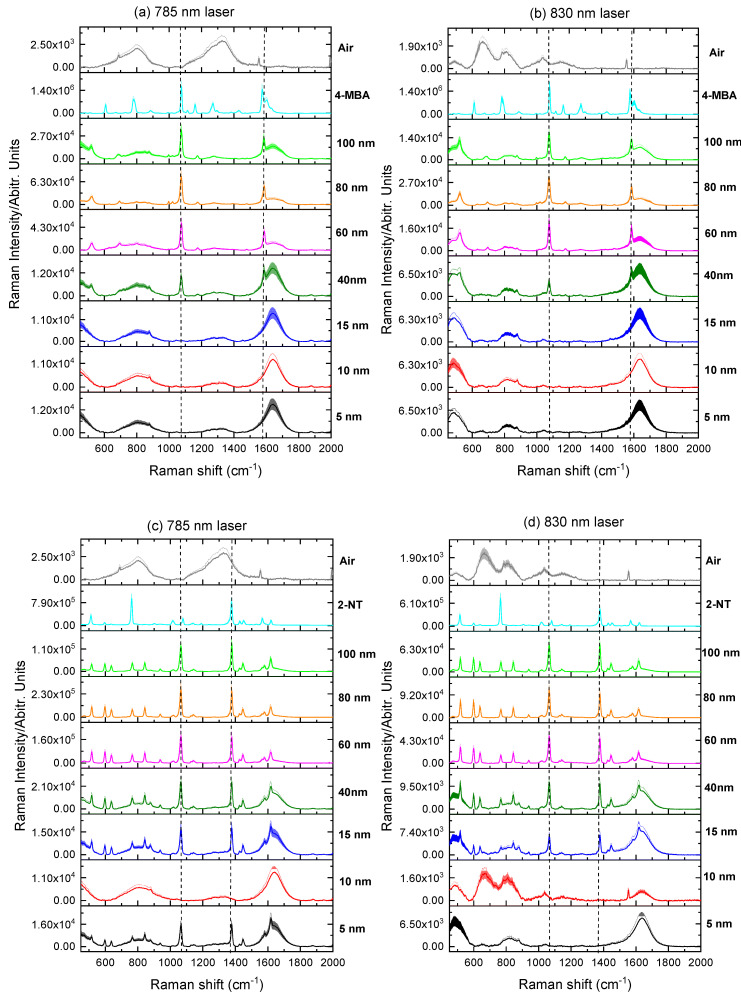
Raman spectra of 4-MBA labeled AuNP solutions measured at (**a**) 785 nm and (**b**) 830 nm; Raman spectra of 2-NT labeled AuNP solutions measured at (**c**) 785 nm and (**d**) 830 nm. Dashed lines denote the two most prominent signals of 4-MBA at 1078 and 1588 cm^−1^. Shading: standard deviation.

**Figure 7:**
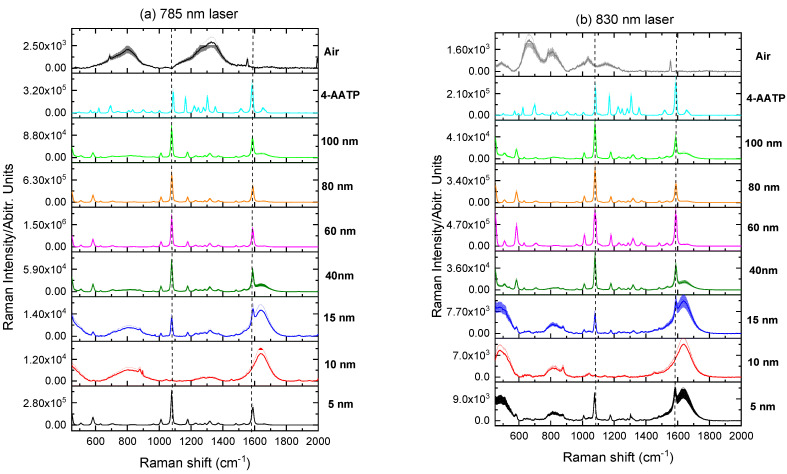
Raman spectra of 4-AATP labeled AuNP solutions measured at (**a**) 785 nm and (**b**) 830 nm.

**Figure 8:**
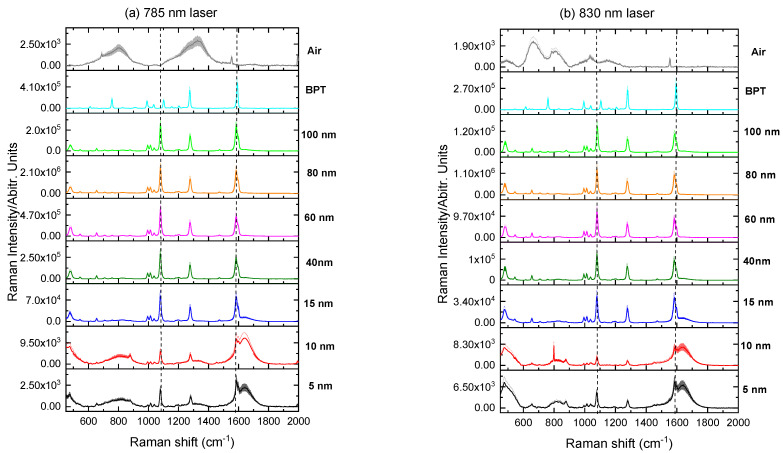
Raman spectra of BPT labeled AuNP solutions measured at (**a**) 785 nm and (**b**) 830 nm. Dashed lines denote the two most prominent signals of BPT at 1080 and 1586 cm^−1^. Shading: standard deviation.

Figure 6, Figure 7 and Figure 8 present the Raman spectra of labeled AuNP solutions prepared using different NP sizes and measured at two wavelengths, 785 nm and 830 nm. For comparison, the spectrum of the pure label (powder) is also shown. Note that there was no Raman signal arising from the AuNPs themselves when reporters were not added spectrum (i.e., NPs blank spectrum). It has been observed that the increase in the maximum absorption wavelength is based on the increasing larger size AuNPs percentage volume. Finally, when only particles with a size of 830 nm are present in the solution, the highest absorption peak is converted to a wavelength of 785 nm. A larger NPs size necessitates such a change. Because it is impossible to see peaks separately from respective populations in these colloids, the maximum location of adsorption of a polydisperse colloid does not provide information about the nanoparticle size.

To assess the SERS signals for each individual label, the two dominant vibrational lines corresponding to the C–H rocking (1080 cm^−1^) and in-plane stretching of the benzene rings (1380 and 1586 cm^−1^) were selected [22].

**Figure 9:**
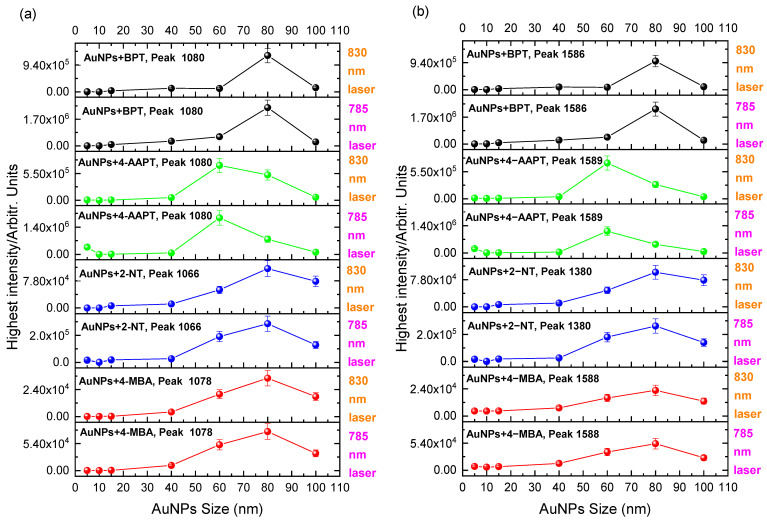
Plot of the intensity (height) of the two main peaks at (**a**) lower and (**b**) higher wavenumbers for labeled AuNP solutions vs. NP size at 785 nm and 830 nm excitation.

As it can be seen from Figure 6, Figure 7 and Figure 8, weak or no signal was observed for labeled AuNPs of sizes smaller than 15 nm. The large signal sometimes observed for 5 nm nanoparticles labeled with 2-NT or 4-AATP could be due to aggregation [23]. Conversely, the signal was strong for AuNPs of size comprised between 40 nm and 100 nm, as shown in Figure 9.

Excitation at 785 nm was found to produce larger signals than those obtained with 830 nm excitation, as it is expected from the wavelength dependence of the scattering intensity [24]. The largest signal was found for BPT labeled 80 nm AuNPs (2.6 × 10^6^) followed by 4-AATP labeled 60 nm AuNPs (2.2 × 10^6^) using the 785 nm laser, suggesting that BPT and 4-AATP are the best candidates for Raman labelling of spherical AuNPs with sizes in the range 5 nm to 100 nm.

## 4. Discussion

This study investigated the physical and spectroscopic properties of commercial spherical gold nanoparticles with diameters ranging between 5 nm and 100 nm in an aqueous solution with four different Raman reporters, 4-MBA, 2-NT, 4-AATP, and BPT chemisorbed on the nanoparticles’ surface. The effect of size is very important for nanoparticle-based SERS active substrates. Therefore, it is essential to select the appropriate NP size and the matching excitation wavelength to excite the localized surface plasmon resonance (LSPR) and obtain the best signal from SERS. Results from UV-visible spectrophotometry showed that 2-NT and BPT labelling produce the largest redshift in LSPR. The surface charge (zeta potential) derived from DLS for the gold nanoparticles before and after adding Raman reporters was negative.

Based on the work of Cyrankiewicz et al. [25] and Haiss et al. [26], the gold nanoparticles’ size and form were determined using the SEM method. The average size of gold nanoparticles with spherical form was determined to be 17, 30, 40, 50, 60, and 80 nm, respectively. Gold NPs with a diameter of 50 nm had the highest standard deviation. The SERS activity and UV-Vis absorption of gold nanoparticles were shown to be stable for at least one month.

The SERS spectra of 4-ATP and 4-NTP were collected using gold NPs of various sizes. The concentration of gold, the number of gold NPs, or the surface area of the gold NPs were all held constant to determine the ideal size of the gold NPs that gives the largest enhancement factor. This phenomenon may be explained by the fact that surface area rises as the size of gold NPs grow while the total number of NPs remains constant, resulting in increased SERS intensity. However, when the overall surface area of gold NPs or the concentration of gold were held constant, fascinating occurrences were observed.

More interestingly, the relationships for 4-ATP and 4-NTP were shown to be the same. This suggests that such findings may not be extremely sensitive to the target molecules’ chemical structure. Instead, the findings might be applied to different adsorbates. When the surface area or concentration is important, gold NPs with a size of roughly 50 nm are ideal. Furthermore, gold nanoparticles with a size of roughly 50 nm have a low cytotoxic effect on biological samples. This conclusion is critical when SERS is used to identify crucial biomolecules in biological samples so that the least amount of gold is injected into the biological system to obtain the lowest toxicity while also achieving the maximum SERS sensitivity.

SERS labels based on noble-metal nanoparticles loaded with Raman-active compounds are promising candidates for ultrasensitive multiplexed assays and in vitro/in vivo imaging due to the surface-enhanced Raman scattering effect. Understanding how to optimize the brightness of such labels, on the other hand, is critical for their broad adoption. It is investigated the effective differential Raman scattering cross-section of SERS labels consisting of pegylated gold nanoparticles loaded with various Raman active chemicals (Raman reporters). It has been discovered that using the right Raman reporter and nanoparticle size may increase the differential Raman scattering cross-section by several orders of magnitude [27]. The experimental results are explained by taking into account the molecular cross-section for resonant Raman scattering and the local electromagnetic enhancement factor in the vicinity of gold nanoparticles. These findings may be used to drive the development of SERS labels with improved performance and as a baseline for comparing the absolute value of the SERS labels based on metal nanoparticles.

Results obtained from Raman measurements on AuNPs with 4-MBA labeling were compared with previous findings on gold nanotips by Gao et al. [28]. The authors have observed the highest SERS signals for tips with 40 nm apex diameter using 4-MBA as the probe molecule and an intensity decrease for tips with 60 and 80 nm. The discrepancy with our data for 4-MBA, which show the strongest signal intensity for gold spheres with 80 nm diameter (Figure 9), is plausibly caused by a difference in curvature and associated molecular packing on the gold substrates.

Even though AgNPs were utilized as SERS substrates in the work, Nabiev et al. [29] published the first report on single-cell SERS investigation on live cells in 1991. It was feasible to distinguish between doxorubicin’s interactions with the cytoplasm and the nucleus. Kneipp et al. [30] used AuNPs as SERS substrates in single living-cell SERS over a decade after this first publication in 2002. Then, until 2010, research concentrated on diverse AuNP applications in single-cell SERS. High-throughput analysis, multiplexed imaging of various biomarkers, theranostic applications, and time-resolved cellular dynamic changes are all hot topics in single-cell SERS right now.

Bare AuNPs can be used to obtain general cellular response signals without organelle-specific targeting. Kuku et al. [31] evaluated the cytotoxic response of cells incubated with different nanomaterials using SERS utilizing 50 nm citrate-reduced spherical AuNPs as SERS substrates in recent research. In the SERS spectra, cell-type, and nanomaterial dose-dependent responses could be plotted. Xu et al. [32] studied the AuNP-AuNR self-assembled structures for possible detection of small molecules such as nitric oxide, glucose, polyamines, and NADPH/NADP+ in HeLa cells in proof-of-concept research. According to the authors, the proposed method may simultaneously identify tiny compounds in a live cell. They claim that the AuNP–AuNR nanostructures may be used as a substitute for immunoassay-based detection of small molecules when one is not available.

Photobleaching, spectrum overlap of various dyes, and chemical instability are all issues with conventional fluorescence-based tags. SERS tags coated with cell-surface biomarkers are regarded as solutions. For example, a SERS tag was created by coating the core of AuNPs (15 nm) with poly adenine to establish a uniform nanogap of 1 nm, which was subsequently decorated with the Raman reporters 4,40-dipyridyl (44DP) and 5,50-dithiobis(2-nitrobenzoicacid) (DTNB) [33]. After that, varying thicknesses of Au shell were applied to the Au core, resulting in NPs with diameters ranging from 40 to 100 nm (Au@Au core-shell). The Au@Au core-shell NPs with the largest improved signal were those with a diameter of 76 nm. The 76 nm Au@Au core-shell NPs were then functionalized with hyaluronic acid (HA) to make them bind to an overexpressed HA receptor biomarker on the cell surface of human colon cancer cells, CD44 protein.

An improved electroporation method was used to circumvent the endolysosomal route as well as the targeting moieties in another AuNP delivery strategy. In this approach, CA46 Burkitt’s lymphoma cells were given Au–Ag core-shell NPs with a Raman reporter, 4-MBA, on the gold core. It was feasible to identify and visualize the distribution of lipids and phenylalanine in the cytoplasm, similar to the small-molecule detection studies published by Xu et al. [32]. As previously stated, the uptake and aggregation of AuNPs in cellular compartments greatly influences the SERS spectra of single cells. Due to differences in intracellular absorption efficiency of AuNPs, the creation of a comparable environment around AuNPs and their aggregates for SERS may not be achievable in each kind of cell [34]. To tackle the issues associated with low repeatability and large variability dependent on intracellular absorption of AuNPs, El-Said et al. [35] created a sensitive SERS-active substrate surface by putting or-dered gold nanodots on an indium tin oxide (ITO) surface. The gold nanodot SERS substrate allowed for cell differentiation, cell cycle phases, and live/dead cell monitoring without compromising the viability of connected cells during sample preparation. The substrates can be used to create a continuous system for time-dependent monitoring in research such as drug development.

## 5. Conclusions

This study shows promising evidence that indicates the surface charged AuNPs through the use of four different Raman nanoparticles. The findings have indicated the absence of a substantial inflammatory activity related to the designed gold nanoparticles, specifically DLS and UV-IVs AuNPs. Therefore, such NPs serve as essential platforms to be used for drug delivery or as ligands in cancer treatment, immunotherapeutic approaches, and chronic infections. Finally, a general challenge in single-cell research, regardless of the applied technique, is the processing of large data sets. Improved algorithms for data reduction have been utilized to overcome this challenge. However, more studies are required to establish a routine analysis of single-cells using SERS. Moreover, future studies should add cell SERS investigation on live cells for determining inflammatory activity.

## Figures and Tables

**Table 1:** Centrifugation rate and time used in the preparation of labeled gold nanoparticle solutions.

AuNP Size (nm)	4-MBA		2-NT		4-AATP		BPT	
	Speed (rpm)	Time (min)	Speed (rpm)	Time (min)	Speed (rpm)	Time (min)	Speed (rpm)	Time (min)
5	10 K	10	4 K	10	4 K	10	10 K	30
10	4 K	10	4 K	10	4 K	10	10 K	30
15	4 K	10	4 K	10	4 K	10	10 K	30
40	3 K	10	3 K	10	3 K	10	4 K	30
60	3 K	10	3 K	10	3 K	10	4 K	30
80	3 K	10	3 K	10	3 K	10	4 K	30
100	3 K	10	3 K	10	3 K	10	4 K	30

## Data Availability

Data is available upon reasonable request.

## Sample Availability

Samples of the compounds are available from the authors.
